# Rationale and design of the optimal antithrombotic treatment for acute coronary syndrome patients with concomitant atrial fibrillation and implanted with new‐generation drug‐eluting stent: OPtimal management of anTIthroMbotic Agents (OPTIMA)‐4 trial

**DOI:** 10.1002/clc.24025

**Published:** 2023-05-16

**Authors:** Xiaoxuan Gong, Rui Hua, Jianling Bai, Tianyu Wu, Qin Wang, Jinhua Zhang, Wenhao Zhang, Lianghong Ying, Yongsheng Ke, Xiaoyan Wang, Xiwen Zhang, Kun Liu, Yan Chen, Boqing Zhang, Peng Dong, Jianqiang Xiao, Changling Li, Li Zhu, Chunjian Li

**Affiliations:** ^1^ Department of Cardiology The First Affiliated Hospital of Nanjing Medical University Nanjing China; ^2^ Department of Biostatistics, School of Public Health Nanjing Medical University Nanjing China; ^3^ The Pharmaceutical Department Fujian Medical University Union Hospital Fuzhou China; ^4^ Department of Cardiology, The Affiliated Huai'an Hospital of Xuzhou Medical University Huai'an Second People's Hospital Huai'an China; ^5^ Department of Cardiology Yijishan Hospital of Wannan Medical College Wuhu China; ^6^ Department of Cardiology The Affiliated Hospital of Jiangnan University Wuxi China; ^7^ Department of Cardiology The Affiliated Huaian No. 1 People's Hospital of Nanjing Medical University Huai'an China; ^8^ Department of Cardiology The First People's Hospital of Lianyungang Lianyungang China; ^9^ Department of Cardiology Taishan People's Hospital Taishan China; ^10^ Department of Cardiology The Second Affiliated Hospital of Nanjing Medical University Nanjing China; ^11^ Department of Cardiology The Affiliated Hospital of Hangzhou Normal University Hangzhou China; ^12^ Department of Cardiology Changzhou Wujin People's Hospital Changzhou China; ^13^ Department of Cardiology, The Second Affiliated Hospital Zhejiang University School of Medicine Hangzhou China; ^14^ Department of Cardiology Taizhou People's Hospital Taizhou China

**Keywords:** acute coronary syndrome, dual antithrombotic treatment, new oral anticoagulants, new‐generation drug‐eluting stent, nonvalvular atrial fibrillation

## Abstract

**Background:**

About 5%–15% of acute coronary syndrome (ACS) patients undergoing stent implantation have concomitant atrial fibrillation and need both antiplatelet and anticoagulant therapies. The optimal antithrombotic regimen remains uncertain in this scenario.

**Hypothesis:**

A multicenter randomized controlled trial (OPtimal management of anTIthroMbotic Agents [OPTIMA]‐4) is designed to test the hypothesis that, for ACS patients with concomitant nonvalvular atrial fibrillation (NVAF) and having low‐to‐moderate risk of bleeding, clopidogrel is comparable in efficacy but superior in safety compared to ticagrelor while being used in combination with dabigatran after new‐generation drug‐eluting stent (DES) implantation.

**Methods:**

ACS patients who have low‐to‐moderate risk of bleeding (e.g., HAS‐BLED score ≤ 2) and require anticoagulation therapy (CHA_2_DS_2_‐VASc score ≥ 2) will be recruited after implantation of new‐generation DES. A total of 1472 eligible patients will be randomly assigned to receive a 12‐month dual antithrombotic treatment of either clopidogrel 75 mg daily or ticagrelor 90 mg twice daily in combination with dabigatran 110 mg twice daily. Participants will be followed up for 12 months after randomization. The primary efficacy endpoint is a composite of cardiovascular death, myocardial infarction, unplanned revascularization, ischemic stroke, and systemic thromboembolism. The primary safety endpoint is set as major bleeding or clinically relevant nonmajor bleeding defined by the International Society of Thrombosis and Hemostasis. The enrollment and follow‐up have been launched.

**Results:**

The first enrollment occurred on March 12, 2018. The recruitment is anticipated to be completed before December 31, 2024.

**Conclusions:**

The OPTIMA‐4 trial offers an opportunity to assess the optimal dual antithrombotic regimen in ACS patients with concomitant NVAF after the implantation of new‐generation DES.

## Graphical abstract

Overview of the design of the OPtimal management of anTIthroMbotic Agents‐4 trial.

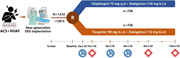

## 1 INTRODUCTION

Dual antiplatelet therapy (DAPT) with aspirin and adenosine diphosphate (ADP) antagonist has been recommended for patients after drug‐eluting stent (DES) implantation to prevent stent thrombosis (recommendation level: IA),^1^ and oral anticoagulants (OAC) is recommended for atrial fibrillation (AF) patients with CHA_2_DS_2_‐VASc score ≥ 2 to prevent ischemic stroke.^2^ Thus, for patients undergoing percutaneous coronary intervention (PCI) with concomitant AF, triple antithrombotic therapy (TATT) including both DAPT and OAC would be indicated.^3^ However, previous studies have reported that TATT based on warfarin or new oral anticoagulants (NOAC) significantly increased the bleeding incidence compared with dual antithrombotic therapy (DATT).^4^, ^5^

The new‐generation DES with better biocompatibility or biodegradable polymers or non‐polymer has been widely used in clinical practice. Compared with the first‐generation DES, the new‐generation DES is associated with faster endothelialization and lower incidence of stent thrombosis and is therefore less dependent on DAPT.^6^ In the era of new‐generation DES, current guidelines have recommended a shortened duration (1 week to 1 month) of TATT in acute coronary syndrome (ACS) patients with concomitant AF undergoing PCI.^2^, ^7^ Additionally, concerning about high bleeding risk prevailing, 12‐month DATT including a NOAC in preference to warfarin is recommended to be administered in ACS patients with concomitant AF after PCI^2^, ^7^ referring to several randomized controlled trials (RCTs), i.e., PIONEER AF‐PCI, RE‐DUAL PCI, AUGUSTUS, and ENTRUST‐AF PCI.^5^, ^8^, ^9^, ^10^ However, it has not been demonstrated which ADP antagonist (e.g., clopidogrel or ticagrelor) is optimal to be coadministered with NOAC. Furthermore, it is uncertain whether DATT is suitable for the same patients but with low to moderate risk of bleeding.

The Platelet Inhibition and Patient Outcomes (PLATO) trial has found that in ACS patients, ticagrelor significantly decreased cardiovascular death by 21.6% compared with clopidogrel while being used in combination with aspirin.^11^ Nevertheless, the ticagrelor arm increased major bleeding by 0.7% (4.5% vs. 3.8%, *p* = .03).^11^ Thus, there is a concern about the bleeding risk under the DATT of NOAC combined with the most potent antiplatelet agent ticagrelor. It is uncertain whether there is any difference between ticagrelor and clopidogrel while being coadministered with a NOAC in post‐PCI ACS patients with concomitant AF.

Our OPtimal management of anTIthroMbotic Agents (OPTIMA)‐4 trial is designed to test the hypothesis that, for ACS patients with concomitant nonvalvular atrial fibrillation (NVAF) and having low‐to‐moderate risk of bleeding, clopidogrel is comparable in efficacy but superior in safety compared to ticagrelor while being used in combination with dabigatran after new‐generation DES implantation.

## 2 METHODS

### 2.1 Study design

**Figure 1:**
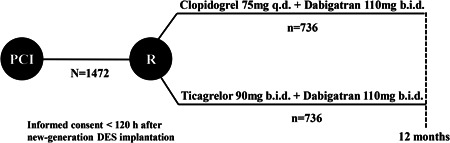
Flowchart of OPtimal management of anTIthroMbotic Agents‐4 trial. DES, drug‐eluting stent; PCI, percutaneous coronary intervention; R, randomization.

A multicenter, open‐label, randomized, controlled clinical trial (OPTIMA‐4) is designed. The study design is shown in Figure 1. The study complies with the Declaration of Helsinki as amended in Seoul, Korea (64th, 2013), and is to be performed in accordance with the International Conference on Harmonization “Good Clinical Practices.” Before participating in this study, patients should be informed about the objectives, rights, duties, possible risks, and benefits of the study in lay language, and written informed consent should be signed as required. Written informed consent for publication of their clinical details and/or clinical images should be obtained from the patient/parent/guardian/relative of the patient. Patients can be identified only by a unique identification code and randomization number to maintain their confidentiality. The investigators will report the progress of this study to the ethics committee annually until the study is completed. The study has been registered at Clinicaltrials.gov (identifier: NCT 03234114).

### 2.2 Participant selection

The main inclusion criteria are ACS patients with concomitant nonvalvular AF, who underwent new‐generation DES implantation with CHA_2_DS_2_‐VASc score ≥ 2 and in an acceptable risk of bleeding (e.g., HAS‐BLED score ≤ 3). The detailed inclusion and exclusion criteria are listed in Table 1. A total of 1472 patients will be required and recruited at 66 medical centers in China (see Supporting Information: Appendix 2).

**Table 1:** Inclusion and exclusion criteria.

Inclusion criteria
≥18 years
ACS patients with concomitant paroxysmal, permanent, or persistent nonvalvular AF underwent new‐generation DES implantation
CHA_2_DS_2_‐VASc score ≥ 2
In an acceptable risk of bleeding at the discretion of researchers (e.g., HAS‐BLED score ≤ 3)
Consent to participate in the trial
Exclusion criteria
DES implanted in the left main coronary artery
Cardiogenic shock or Killip III–IV
STEMI patients with malignant arrhythmias or underwent electrodefibrillation or cardiopulmonary resuscitation or with cardiac mechanical complications (heart rupture, ventricular septal perforation, nipple muscle fracture, etc.)
History of gastrointestinal or intracranial hemorrhage; active bleeding, trauma, or major surgery within 1 month; suspected or diagnosed aortic dissection
Ischemic stroke with limb dysfunction or dysphasia
Known allergy or intolerance to the study medications: warfarin, clopidogrel, aspirin, dabigatran, ticagrelor, and heparin
Participating in other ongoing trials
Planned surgery in 12 months requiring to withdraw of the antiplatelet agents
Planned AF ablation or left atrial appendage occlusion in the next 12 months
Abnormal liver or kidney function (ALT > 3 ULN; estimated CrCl < 30 mL/min/1.73 m^2^ calculated by Cockcroft–Gault equation); diagnosed of liver cirrhosis
Hematological disease with bleeding tendency; hemoglobin < 100 g/L; platelet count < 100 × 10^9^ cells/L
Malignancies or life expectancy of less than 1 year
Pregnant (present, suspected, or planned) or lactating woman
Patients who are taking drugs which may interact with study agents, such as miconazole, ketoconazole, fluconazole, voriconazole, itraconazole, posaconazole, efinaconazole, and rifampicin
Patients with any other conditions that may not be suitable to participate in the trial at the discretion of the researchers

Abbreviations: ACS, acute coronary syndrome; AF, atrial fibrillation; ALT, alanine aminotransferase; CrCl, creatinine clearance; DES, drug‐eluting stent; STEMI, ST‐segment elevation myocardial infarction; ULN, upper limit of normal.

### 2.3 Randomization

Within 120 h of the new‐generation DES implantation, eligible patients will be randomized in a 1:1 ratio to receive either clopidogrel 75 mg daily or ticagrelor 90 mg twice daily in combination with dabigatran 110 mg twice daily for 12 months. The randomization will be done by the investigators through a central 24 h computerized randomization system of REDCap,^12^ using a random permuted block design with block sizes of four. The system can be accessed using personalized login data. Treatment allocation will be revealed for the patients and attending physicians.

### 2.4 PCI procedure

PCI procedures will be performed by well‐experienced interventional cardiologists performing over 500 cases of PCI per year, and radial access should be chosen as a priority. Successful PCI is defined as thrombolysis in myocardial infarction flow grade 3 in the target vessel with less than 10% residual stenosis. New‐generation DES (with better biocompatibility or biodegradable polymers or non‐polymer demonstrated in Supporting Information: Appendix 3) is required to be implanted in OPTIMA‐4 trial.

### 2.5 Combined medications

The low‐molecular‐weight heparin (LWMH) (100 IU/kg, injected subcutaneously every 12 h) is recommended for up to 7 days during the perioperative period. Dabigatran is required to be prescribed after randomization and discontinuation of the LWMH. A bolus of unfractionated heparin (UFH) (100 IU/kg) should be injected intravenously during the PCI procedure. The activated clotting time (ACT) should be monitored every 1 h and an extra UFH should be administered if the ACT is low than 300 s. The platelet glycoprotein IIb/IIIa receptor inhibitors, e.g., Tirofiban, are not recommended unless a heavy thrombus burden is shown in coronary angiography. Other antithrombotic agents, e.g., prasugrel or rivaroxaban, are not allowed to be administered during the study period. Proton pump inhibitors could be administered for patients with high risk of gastrointestinal bleeding. Other medications (e.g., statins, β‐blockers, angiotensin‐converting enzyme inhibitors, etc.) could be administered at the discretion of the attending physicians.

### 2.6 Pharmacodynamic, pharmacokinetic, and pharmacogenomic studies

The pharmacodynamic, pharmacokinetic, and pharmacogenomic studies of the antithrombotic agents will be performed in qualified centers for consented participants. The pharmacodynamic study of the antiplatelet drugs will be performed by light transmission aggregometry (LTA). In detail, 2 mL venous blood will be separately collected into two 3.2% sodium citrate vacutainer tubes (Becton, Dickinson and Company) at 7:30 a.m. on the day of discharge. Then blood samples will be subjected to an LTA test within 2 h, as previously described.^13^ Samples will be centrifuged at 200*g* for 8 min to obtain platelet‐rich plasma (PRP). Platelet‐poor plasma (PPP) will be prepared by centrifuging the remaining blood at 2465*g* for 10 min. Platelet counts will be adjusted by adding PPP to the PRP to achieve a count of 250 × 10^9^/L. Both the ADP (final concentration 5 μmol/L) induced platelet aggregation (PL_ADP_) and the arachidonic acid (AA) (final concentration 1 mmol/L) induced platelet aggregation (PL_AA_) will be detected using the Chronolog Model 700 aggregometer (Chrono‐log Corporation). The pharmacokinetic study of dabigatran will be performed as follows. Venous blood (2 mL) will be collected into a 3.2% sodium citrate vacutainer tube (Becton, Dickinson and Company) at 0.5 h before the dosing after at least six doses. Samples will be centrifuged at 2500*g* for 15 min at 25°C within 1 h. After centrifugation, the upper plasma will be stored below −80°C, and the concentration of dabigatran will be detected by high‐performance liquid chromatography‐tandem mass spectrometry in the pharmaceutical department of Fujian Medical University Union Hospital.^14^ The pharmacogenomic studies of the antithrombotic agents will be performed as follows. Two milliliters of blood will be drawn into a BD Vacutainer tube (Becton, Dickinson and Company) containing 3.6 mg K2‐ethylenediaminetetraacetic acid and stored below −80°C. The following single‐nucleotide polymorphisms (SNPs) will be detected: (1) clopidogrel‐related SNPs: CYP2C19 (rs12248560, rs28399504, rs41291556, rs4244285, rs4986893, rs5633701, rs72552267, rs72558186)^15^, ^16^; (2) ticagrelor‐related SNPs: SLCO1B1 (rs113681054), OATP1B1 (rs4149056), CYP3A4 (rs62471956, rs56324128), UGT2B7 (rs61361928)^17^; and (3) dabigatran‐related SNP: ABCB1 (rs4148738, rs1045642), CES1 (rs8192935).^18^, ^19^

### 2.7 Endpoints

The primary efficacy endpoint of the OPTIMA‐4 trial is a composite of cardiovascular death, myocardial infarction, unplanned revascularization, ischemic stroke, and systematic thromboembolism during 12‐month follow‐up. The primary safety endpoint is major bleeding and clinically relevant nonmajor bleeding (CRNMB) defined by the International Society of Thrombosis and Hemostasis (ISTH) during 12‐month follow‐up.^20^ The composite secondary endpoint of net adverse events included all‐cause death (cardiovascular death, noncardiovascular death), acute myocardial infarction (fatal or nonfatal, Q‐wave or non‐Q‐wave), systemic thromboembolism, unplanned revascularization (target or nontarget vessel, target or nontarget lesion), stent thrombosis (possible, probable, definite), stroke (hemorrhage or ischemic) and bleeding (ISTH or Bleeding Academic Research Consortium [BARC] criteria) during 12‐month follow‐up.^20^, ^21^

Cardiovascular deaths include death from myocardial infarction, stroke, heart failure, aortic dissection, cardiac arrest (sudden death), cardiovascular operations, and unknown causes for which investigators fail to acquire any relevant information in the follow‐up.^21^ Myocardial infarction is defined in accordance with the fourth universal definition (types 1–5).^22^ Unplanned revascularization consists of unplanned PCI or surgical bypass for a >70% stenosis by coronary angiography with ischemic symptoms or a >90% stenosis with or without symptoms. Target vessel revascularization is defined as the revascularization of any segment of the target vessel. Target lesion revascularization is defined as revascularization for the target lesion or bypass surgery of the target vessel performed for restenosis or other complication of the target lesion. Stent thrombosis is defined referring to the criteria of the Academic Research Consortium.^23^ Stroke is defined as the presentation of acute vasogenic focal neurological defect with relevant symptoms and signs lasting ≥24 h, which should be confirmed by computed tomography or magnetic resonance imaging and categorized into a hemorrhagic stroke, ischemic stroke, subarachnoid hemorrhage, or undetermined stroke.^21^ Systemic thromboembolism is defined as an acute limb or organ (kidney, mesenteric artery, spleen, retina, or graft) vascular obstruction determined by imaging, surgery, or autopsy, with or without corresponding symptoms or signs.^23^ Bleeding is classified as (1) major bleeding, CRNMB, and minor bleeding by ISTH criteria; (2) types 1–5 with major bleeding ≥type 3a according to BARC criteria.^20^, ^21^ The detailed definitions of endpoints are summarized in Supporting Information: Appendix 4. All primary endpoints will be evaluated by an independent events review committee (ERC) blinded to treatment assignment.

### 2.8 Study visits and follow‐up

All subjects will be followed up at 1 month (±7 days), 6 months (±7 days), and 12 months (±7 days) in the outpatient clinic, and at 2 weeks (±7 days), 2 months (±7 days), and 3 months (±7 days) by telephone. The patients' primary and secondary endpoints, (severe) adverse events, medications, and the results of the tests mentioned above will be carefully recorded during the follow‐up.

### 2.9 Sample size determination and statistical analysis

The OPTIMA‐4 trial aims to simultaneously test the following two hypotheses of dabigatran‐based DATT in eligible patients: (1) clopidogrel is superior to ticagrelor regarding major bleeding and CRNMB events and (2) clopidogrel is noninferior to ticagrelor regarding the composite efficacy endpoint. According to the RE‐DUAL PCI trial, the anticipated incidence of annual efficacy endpoint is 18.9% in the arm of ticagrelor.^9^ We set a noninferiority margin ratio of 1.38 and *α* of .025 (one‐sided), 655 patients in each group are required to achieve a statistical power of 80%.^9^ Assuming that 11% of patients will be lost to follow‐up (1%) or drop‐out (10%), at least 736 patients are finally required for each randomized group. Then, the final sample size is estimated to be 1472. With this sample size, the anticipated power for the composite safety endpoints (14.5% and 21.2% in clopidogrel and ticagrelor groups, respectively) would be around 87.1%.^9^

Descriptive statistics will be used to summarize and analyze the baseline demographic characteristics (mean, SD, median, minimum, first quartile, third quartile, and maximum for continuous measures; frequency counts and percentages for categorical measures) of each treatment group; the Student *t* test or analysis of variance will be used for the comparison of continuous variables and *χ* ^2^ test for the comparison of categorical variables across treatment groups.

To control the type I error at one‐side 0.025 level, a hierarchical procedure for multiple testing will be used. The paradigm of the hierarchical procedure in OPTIMA‐4 trial is detailed below:

Step 1: Superiority of dabigatran combined with clopidogrel to dabigatran combined with ticagrelor concerning major bleeding and CRNMB is met at the one‐sided 0.025 level of significance.
Step 2: Noninferiority of dabigatran combined with clopidogrel to dabigatran combined with ticagrelor for the efficacy endpoint is met at the one‐sided 0.025 level of significance.

If step 1 fails to meet statistical significance, step 2 will not be performed.

The primary endpoints will be treated as time‐to‐event endpoints, and the Kaplan–Meier method will be employed to estimate the cumulative incidences of endpoints by treatment group. The primary endpoints between the two groups will be compared using the Cox proportional hazards model with treatment as a fixed effect and adjusted for other covariates separately. The estimated hazard ratio (HR) and two‐sided 95% confidential interval (CI) or one‐sided 97.5% CI for the noninferiority test will be provided. For the noninferiority test in the composite efficacy endpoint, the noninferiority margin is 1.38 on the HR scale. The upper boundary of CI for the HR (one‐sided 97.5%) will be compared to this margin. All the above analyses will also be conducted in predefined subgroups. The secondary time‐to‐event endpoints will be analyzed using the methods mentioned above. All statistical analyses will be performed using SAS version 9.4 (SAS Institute Inc.).

The analysis sets are as follows: (1) full analysis set (FAS): according to the intention‐to‐treat principle, the FAS is defined as all patients being randomized who receive at least one dose of the assigned treatment; (2) per‐protocol analysis set (PPS): the PPS is a subset of FAS, which includes all randomized patients meeting the study eligibility criteria, without major protocol deviation (see Supporting Information: Appendix 5),^21^, ^24^ without using drugs or receiving treatments that may interfere with the evaluation of efficacy, and accomplishing all efficacy assessments after randomization; and (3) safety set (SS): the SS includes all randomized patients with at least one safety assessment after randomization. The analysis of primary endpoints will be performed on both FAS and PPS, with FAS analysis as the primary analysis. Consistency of the results on primary endpoints between FAS and PPS will demonstrate the robustness of the conclusion. Safety analyses will be performed on the SS.

Subgroup analyses are planned across major subgroups by age, sex, body mass index, diabetes, ACS diagnosis, the Synergy between Percutaneous Coronary Intervention with Taxus and Cardiac Surgery (SYNTAX) score, and CHA_2_DS_2_‐VASc score (see Supporting Information: Appendix 6).

### 2.10 Study administration and management

The local Institutional Review Board or Ethics Committee at each participating co‐center must approve the study, and all patients must provide written informed consent before enrollment. Funding will be provided by the Department of Cardiology, the First Affiliated Hospital of Nanjing Medical University for the co‐centers. The First Affiliated Hospital of Nanjing Medical University will preserve the complete study database and perform all key analyses. The Steering Committee, independent Data Safety Monitoring Board (DSMB), and ERC have been set for the safety control and the evaluation of the primary endpoints (see Supporting Information: Appendix 1). The DSMB may make recommendations to the Steering Committee and study sponsor as a result of its monitoring activities.

## 3 DISCUSSION

OPTIMA‐4 trial is the first large‐scale RCT investigating the optimal P2Y_12_ inhibitor in combination with dabigatran for ACS patients with concomitant NVAF undergoing new‐generation DES implantation. This trial will add new evidence on the optimal antithrombotic strategy for the above mentioned patients.

TATT was proposed for ACS patients with concomitant AF after PCI to prevent major adverse cardiovascular events (MACEs) over a decade ago.^25^ Nowadays, the ischemic risk for these patients has been reduced due to the improvement of new‐generation DES and the introduction of advanced antithrombotic agents,^11^, ^26^, ^27^ while the bleeding risk becomes prevailing for these patients under TATT.^28^ As a result, DATT has been recommended as the default strategy for non‐ST‐segament elevation ACS patients requiring chronic OAC, using a NOAC and a single oral antiplatelet agent (preferably clopidogrel) for 12 months (recommendation level: IA) based on large‐scale RCTs.^7^ Anyhow, evidence of the optimal DATT for these patients is limited. Thus, our OPTIMA‐4 trial is designed to compare clopidogrel with ticagrelor in dabigatran‐based DATT for ACS patients with concomitant NVAF and undergoing new‐generation DES implantation, by which we aim to provide evidence for future guidelines in determining the optimal P2Y_12_ inhibitor for these patients.

PLATO trial demonstrated that for ACS patients treated with clopidogrel plus aspirin, the risk of major bleeding not related to coronary‐artery bypass grafting was significantly decreased compared with those treated with ticagrelor plus aspirin.^11^ Thus, it is probable that clopidogrel is superior to ticagrelor in dabigatran‐based DATT with respect to safety. On the other hand, the real‐world evidence showed that in Chinese ACS patients who underwent new‐generation DES implantation, clopidogrel was comparable to ticagrelor in aspirin‐based DAPT in reducing the incidence of MACEs.^29^ Therefore, clopidogrel could be noninferior to ticagrelor in combination with dabigatran concerning the efficacy in ACS patients with concomitant NVAF. In OPTIMA‐4 trial, we presume that for ACS patients with concomitant NVAF and implanted with new‐generation DES, clopidogrel is superior to ticagrelor regarding the safety, and noninferior to the latter regarding the efficacy when coadministered with dabigatran.

To evaluate the benefit‐risk balance, the primary endpoints of OPTIMA‐4 trial contain both safety and efficacy endpoints. The primary safety endpoint is set as major bleeding and CRNMB, which is consistent with the relevant RCTs,^5^, ^8^, ^9^, ^10^ and the primary efficacy endpoint in this trial includes both the cardiocerebrovascular and the peripheral vascular events. The anticipated incidence of major bleeding and CRNMB in each group and noninferiority margin ratio of efficacy endpoint are referred to the RE‐DUAL PCI trial, which contained DATT of dabigatran and a P2Y_12_ inhibitor of either clopidogrel or ticagrelor.^9^

The hierarchical statistical procedure is first based on the hypotheses that the clopidogrel group is superior to the ticagrelor group regarding the safety endpoint, and then noninferior to the latter regarding the efficacy endpoint. This design mainly concerns the major bleeding and CRNMB under DATT, which may result in unexpected discontinuation of antithrombotic agents and consequent increase of the ischemic risk.

The main inclusion and exclusion criteria are set on the basis of the following considerations. First, as only new‐generation DES is required to be implanted in this trial, and the high thrombotic risk scenario such as left main coronary artery DES implantation will be excluded, it would be acceptable to initiate DATT within 5 days after PCI, instead of after 1‐week  1‐month TATT. Second, because all enrolled patients will receive dabigatran‐based DATT, they need to meet the criteria of CHA_2_DS_2_‐VASc score ≥ 2 which indicates high risk of thromboembolism and requirement of anticoagulant. Third, patients with acceptable risk of bleeding (e.g., HAS‐BLED score ≤ 3) will be recruited, whereas those with high risk of bleeding will be excluded, considering the fact that 12‐month DATT has been recommended by current guidelines for the latter.^7^ Besides, previous studies enrolled patients with the average HAS‐BLED score ≤ 3 and showed the superiority of 12‐month NOAC‐based DATT.^5^, ^8^, ^9^, ^10^ Finally, valvular AF patients will not be enrolled owing to little evidence that NOAC is beneficial for these patients.^30^

## AUTHOR CONTRIBUTIONS

Chunjian Li and Xiaoxuan Gong designed the study. Xiaoxuan Gong, Rui Hua, and Jianling Bai contributed equally to writing the manuscript and share the first authorship. Jianling Bai made the statistical analysis plan and Jinhua Zhang provided the pharmacokinetics detection of the antithrombotic agents. Other authors were major contributors to patients' enrollment and management from co‐centers. All authors have read and approved the final manuscript.

## CONFLICT OF INTEREST STATEMENT

The authors declare no conflict of interest.

## Supporting information

Additional supporting information can be found online in the Supporting Information section at the end of this article.

## Data Availability

Data sharing is not applicable to this article now as patient enrollment is not finished yet.
