# Supplementary material for: Rationale and design of the optimal antithrombotic treatment for acute coronary syndrome patients with concomitant atrial fibrillation and implanted with new‐generation drug‐eluting stent: OPtimal management of anTIthroMbotic Agents (OPTIMA)‐4 trial
Source: Clin Cardiol. 2023 May 16;46(7):777–84. doi: 10.1002/clc.24025 (PMC10352971; doi:10.1002/clc.24025)
Supplement: Supplementary file 1 — Additional supporting information can be found online in the Supporting Information section at the end of this article. [file CLC-46-777-s001.docx]

**Appendix 1. Committees of OPTIMA-4 trial**

**Steering committee:** Gregory Y H Lip (Liverpool Centre for Cardiovascular Science, University of Liverpool and Liverpool Heart & Chest Hospital, Liverpool, UK), John W Eikelboom (McMaster University, Hamilton General Hospital, Hamilton, Canada), Hongbing Shen (National Bureau of Disease Control and Prevention, Beijing, China)**,** Jun Zhu (Fuwai Hospital and National Center for Cardiovascular Diseases, Beijing, China)**,** Jun Huang (The First Affiliated Hospital of Nanjing Medical University, Jiangsu, China), Xiangqing Kong (The First Affiliated Hospital of Nanjing Medical University Jiangsu, China), Hao Yu (Nanjing Medical University, Jiangsu, China)

**Data Safety Monitoring Board (DSMB):** Xiuqing Wang (International Cooperation, The First Affiliated Hospital of Nanjing Medical University, Jiangsu, China), Fuming Zhang (Cardiology, The First Affiliated Hospital of Nanjing Medical University, Jiangsu, China), Wenxi Wu (General surgery, The First Affiliated Hospital of Nanjing Medical University, Jiangsu, China), Ting Wu (Neurology, The First Affiliated Hospital of Nanjing Medical University, Jiangsu, China), Lijuan Chen (Hematology, The First Affiliated Hospital of Nanjing Medical University, Jiangsu, China), Jianling Bai (Epidemiology and Biostatistics, Nanjing Medical University, Jiangsu, China)

**Events Review Committee (ERC):** Jun Zhu (Cardiology, Fuwai Hospital and National Center for Cardiovascular Diseases, Chinese Academy of Medical Sciences and Peking Union Medical College, Beijing, China), Xinli Li (Cardiology, The First Affiliated Hospital of Nanjing Medical University, Jiangsu, China), Ting Wu (Neurology, The First Affiliated Hospital of Nanjing Medical University, Jiangsu, China), Jianqing Ge (Neurology, Nanjing Brain Hospital Affiliated with Nanjing Medical University, Jiangsu, China), Chengchun Tang (Interventional Cardiology, Zhong-Da Hospital Affiliated with South-East University, Jiangsu, China), Jie Song (Interventional Cardiology, Gu-Lou Hospital Affiliated with Nanjing University, Jiangsu, China), Mingfang Li (Cardiology, The First Affiliated Hospital of Nanjing Medical University, Jiangsu, China)

**Appendix 2. Centers and investigators in OPTIMA-4 trial**

1. The First Affiliated Hospital of Nanjing Medical University, Jiangsu, China (PI: Chunjian Li; Sub-I: Xiaoxuan Gong)
2. Huai’an First People’s Hospital, Jiangsu, China (PI: Xiwen Zhang; Sub-I: Jing Wang)
3. People’s Hospital of Fuping County, Shanxi, China (PI: Ruina Hao; Sub-I: Cheng Peng)
4. The Second People’s Hospital of Huai’an, Jiangsu, China (PI: Naiquan Yang; Sub-I: Lianghong Ying)
5. Changzhou Wujin People’s Hospital, Jiangsu, China (PI: Jianqiang Xia; Sub-I: Lei Gao)
6. The Second Affiliated Hospital, Zhejiang University School of Medicine, Zhejiang, China (PI: Jun Jiang; Sub-I: Heyang Wang)
7. Affiliated Hospital of Weifang Medical University, Shandong, China (PI: Jingtian Li; Sub-I: Tao Huang)
8. The Second People’s Hospital of Lianyungang, Jiangsu, China (PI: Shuhua Zhang, Liming Sun, Yilian Wang; Sub-I: Zuncai Gu)
9. Hongze Huai’an District People’s Hospital, Jiangsu, China (PI: Xiangshe Wei)
10. Affiliated Hospital of Jiangnan University, Jiangsu, China (PI: Xiaoyan Wang; Sub-I: Jingjing Shi)
11. Taishan People’s Hospital, Guangdong, China (PI: Yan Chen; Sub-I: Haijian Chen)
12. Suzhou Municipal Hospital, Jiangsu, China (PI: Jun Zhang; Sub-I: Junrong Gong)
13. The Second Affiliated Hospital with Nanjing Medical University, Jiangsu, China (PI: Boqing Zhang; Sub-I: Juan Li)
14. Zaozhuang Municipal Hospital, Shandong, China (PI: Jun Yang; Sub-I: Guiying Yan)
15. Aviation Hospital, Beijing, China (PI: Peng Dong; Sub-I: Lianfeng Liu)
16. The Affiliated Suqian First People’s Hospital of Nanjing Medical University, Jiangsu, China (PI: Zhiyong Zhang; Sub-I: Yi Cheng)
17. Nanjing Pukou Hospital of Traditional Chinese Medicine, Jiangsu, China (PI: Chunling Wang; Sub-I: Wenchao Pan)
18. Nantong Tongzhou People’s Hospital, Jiangsu, China (PI: Yuanzhou Lu; Sub-I: Wei Gong)
19. Pukou Branch of Jiangsu Province Hospital, Jiangsu, China (PI: Li Wang; Sub-I: Weiwei Chong)
20. The First People’s Hospital of Lianyungang, Jiangsu, China (PI: Bo Zhao; Sub-I: Mingzhu Li)
21. Yijishan Hospital of Wannan Medical College, Anhui, China (PI: Yongsheng Ke; Sub-I: Yang Ling)
22. The 2nd Affiliated Hospital and Yuying Children’s Hospital of Wenzhou Medical University, Zhejiang, China (PI: Xueqiang Guan; Sub-I: Jinsheng Wang)
23. Jiangsu Taizhou People’s Hospital, Jiangsu, China (PI: Li Zhu; Sub-I: Ruzhu Wang)
24. Changzhou No.2 People’s Hospital, Jiangsu, China (PI: Xin Chen; Sub-I: Jingcheng Chen)
25. The Second Hospital of Nanjing, Jiangsu, China (PI: Xiaofeng Zhang)
26. The People’s Hospital of Liaoning Province, Liaoning, China (PI: Bo Luan; Sub-I: Yong Wang)
27. The First Hospital of Dalian Medical University, Liaoning, China (PI: Bo Zhang; Sub-I: Yajuan Lin)
28. The Second Hospital of Dalian Medical University, Liaoning, China (PI: Xin Zhao; Sub-I: Chuan Lu)
29. Xuyi Hospital of Traditional Chinese Medicine, Jiangsu, China (PI: Yanhong Liu; Sub-I: Zishang Geng)
30. The Affiliated Hospital of Xuzhou Medical University, Jiangsu, China (PI: Yuan Lu; Sub-I: Zhi Li)
31. Northern Jiangsu Province Hospital, Jiangsu, China (PI: Shenghu He; Sub-I: Jun Ji)
32. Xuzhou Third People’s Hospital, Jiangsu, China (PI: Yaojun Zhang; Sub-I: Changsheng Neng)
33. West China Hospital of Sichuan University, Sichuan, China (PI: Yong He; Sub-I: Zhongxiu Chen)
34. Yancheng Third People’s Hospital, Jiangsu, China (PI: Bin Wang)
35. BenQ Hospital of Nanjing Medical University, Jiangsu, China (PI: Hao Ding; Sub-I: Jinshuang Li)
36. Jurong People Hospital, Jiangsu, China (PI: Jun Liu; Sub-I: Xiuli Wu)
37. Affiliated Hospital of Nantong University, Jiangsu, China (PI: Hongzhuan Sheng; Sub-I: Xiaofei Li)
38. The Central Hospital of Taiyuan City, Shanxi, China (PI: Dengfeng Ma; Sub-I: Xin Su)
39. The First Affiliated Hospital of Nanchang University, Jiangxi, China (PI: Xiaoping Peng; Sub-I: Jinghai Hua)
40. Suqian People’s Hospital of Nanjing Drum-tower Hospital Group, Jiangsu, China (PI: Xin Li; Sub-I: Jinmin Zheng)
41. Xuzhou Central Hospital, Jiangsu, China (PI: Yijie Huang, Wen Lu; Sub-I: Pengsheng Chen, Zhen Chen)
42. Women and Children Branch Hospital of Jiangsu Province Hospital, Jiangsu, China (PI: Dianfu Li; Sub-I: Junhong Wang)
43. The First Affiliated Hospital with Anhui Medical University, Anhui, China (PI: Xianhe Lin; Sub-I: Yimin Zhang)
44. Changzhou Hospital of Traditional Chinese Medicine, Jiangsu, China (PI: Xiaolong Li; Sub-I: Yuqiao Chen)
45. Renji Hospital of Shanghai Jiaotong University School of Medicine, Shanghai, China (PI: Jun Bu; Sub-I: Gao Ding)
46. Shengjing Hospital of China Medical University, Liaoning, China (PI: Tiesheng Niu; Sub-I: Jie Chen)
47. The First People’s Hospital of Nantong, Jiangsu, China (PI: Yefei Li)
48. The Second Affiliated Hospital Zhejiang University School of Medicine Changxing Campus, Zhejiang, China (PI: Shiqiang Li; Sub-I: Xue Wang)
49. Yancheng City No.1 People’s Hospital, Jiangsu, China (PI: Jinlong Zhang, Zhihua Wang; Sub-I: Longjin Chen)
50. Suqian Zhongwu Hospital, Jiangsu, China (PI: Yong Cheng; Sub-I: Mingming Zhang)
51. People’s Hospital of Ningxia Hui Autonomous Region, Ningxia, China (PI: Shujun Wei; Sub-I: Shuping Gao)
52. The Third Xiangya Hospital of Central South University, Hunan, China (PI: Yu Cao; Sub-I: Huiling Zhou)
53. Xinghua City People’s Hospital, Jiangsu, China (PI: Bingnan Yao; Sub-I: Yinjun Zhu)
54. The First Affiliated Hospital of Air Force Medical University, Shanxi, China (PI: Ling Tao, Tao Hu; Sub-I: Ruining Zhang)
55. Shanghai No.6 People’s Hospital, Shanghai, China (PI: Chengxing Shen; Sub-I: Hong Shen)
56. Wenzhou Central Hospital, Zhejiang, China (PI: Wenbing Jiang; Sub-I: Yunlong Wang)
57. The First People’s Hospital of Changzhou, Jiangsu, China (PI: Xiaoyu Yang; Sub-I: Sheng Zhang)
58. Affiliated Hospital of Yangzhou University, Jiangsu, China (PI: Chen Liu)
59. Zhenjiang First People’s Hospital, Jiangsu, China (PI: Chunyang Yin)
60. Sir Run Run Hospital, Nanjing Medical University, Jiangsu, China (PI: Daimin Zhang; Sub-I: Lei Xu)
61. The Affiliated Hospital of Hangzhou Normal University, Zhejiang, China (PI: Peng Dong; Sub-I: Hangzhou Luo)
62. Nanjing Qixia District Hospital, Jiangsu, China (PI: Daxing Yang)
63. Nanjing Tongren Hospital, Jiangsu, China (PI: Jiakuan Wu)
64. The Affiliated Xuancheng Hospital of Wannan Medical College, Anhui, China (PI: Long Hu)
65. Shenzhen People's Hospital, Guangdong, China (PI: Da Yin)
66. Wuxi No.2 People’s Hospital, Jiangsu, China (PI: Yan Jin; Sub-I: Qiang Huang)

Abbreviations: PI, principal investigator; Sub-I, sub-investigator.

**Appendix 3. Types and properties of new-generation DES used in OPTIMA-4 trial**

1. Stent name: Xience V, Xience Prime, Endeavor Resolute, Resolute Intergrity, Firebird, Firebird 2, Firekingfisher, Partner

Stent structure: cobalt chromium or cobalt nickel alloy

Polymer property: better biocompatibility

Coating drug: everolimus or zotarolimus or sirolimus

1. Stent name: Synergy, BioMatrix, Nobori, Ultimaster, Orsiro, Promus Premier, EXCEL, EXCROSSAL, FireHawk, BuMA, Helioos, Cordimax, TIVOLI, Noya, GuReater, NOYA

Stent structure: cobalt chromium or platinum chrome or stainless steel

Polymer property: biodegradable

Coating drug: everolimus or sirolimus

1. Stent name: Yukon, Cre 8, BioFreedom, NANO plus, Yinyi

Stent structure: cobalt chromium or stainless steel

Polymer property: polymer-free

Coating drug: everolimus or sirolimus or paclitaxel

Abbreviation: DES, drug-eluting stent.

**Appendix 4. Definitions of endpoints in OPTIMA-4 trial**

**Efficacy endpoints**

**Ⅰ. Death**

1. **Cardiovascular death ^1^**

Death caused by cardiovascular diseases including death from myocardial infarction, stroke, heart failure, aortic dissection, cardiac arrest (sudden death), cardiovascular operations, etc. Death from unknown cause when investigators fail to acquire any relevant information in the follow-up belongs to cardiovascular death. Death caused by complications of cardiovascular events (such as pneumonia after myocardial infarction or stroke) within 30 days of the original event also belong to cardiovascular death.

1. **Definite cardiovascular death**

Death with a clear relation to cardiovascular disease documented in the original medical records is defined as definite cardiovascular death.

1. **Possible cardiovascular death**

Death with a clear relation to cardiovascular disease but without reliable medical record to support it (e.g. diagnosis/death certificate only), or death with an unclear relation to the cardiovascular cause but without definite non-cardiovascular cause, or death from unknown cause is defined as possible cardiovascular death.

1. **Major bleeding death ^2^**

Death caused by direct or indirect (such as hemostatic surgery) major bleeding defined in types 3, 4, and 5 in the Bleeding Academic Research Consortium (BARC) criteria. Death caused by hemorrhagic stroke belongs to major bleeding death and also to stroke endpoints, thus investigators need to report both a major bleeding event and a stroke event. Death caused by ischemic events due to discontinuation of antithrombotic drugs after bleeding does not belong to major bleeding death and the corresponding ischemic events should be reported.

1. **Definite major bleeding death**

Death with a clear relation to major bleeding documented in original medical records is defined as definite major bleeding death.

1. **Possible major bleeding death**

Death with a clear relation to major bleeding but without reliable medical records to support it (e.g. diagnosis/death certificate only), or death with an unclear relation to major bleeding but without other cause is defined as possible major bleeding death.

1. **Non-cardiovascular death**

Death caused by a non-cardiovascular condition.

1. **Definite non-cardiovascular death**

Death with a clear relation to a non-cardiovascular condition documented in the original medical records is defined as definite non-cardiovascular death.

1. **Possible non-cardiovascular death**

Death with a clear relation to a non-cardiovascular condition but without reliable medical records to support it (e.g. diagnosis/death certificate only), or death with an unclear relation to a non-cardiovascular condition but without other cause is defined as possible non-cardiovascular death.

**Ⅱ. Myocardial infarction ^2^**

Myocardial infarction is defined as ≥1 type of cTn values above the 99th percentile upper reference of limit (URL) and presentation of ≥ 1 of the following myocardial ischemic manifestations. Cardiovascular death event should be reported in the meantime when a patient dies of myocardial infarction.

1. Symptoms of typical ischemic chest pain or tightness or constriction, etc. which last more than 20 minutes;
2. New ischemic electrocardiogram (ECG) changes including new ischemic ST depression or elevation, new T-wave inversion and new left bundle branch block (LBBB);
3. New development of pathological Q wave;
4. New imaging evidence of myocardial ischemia;
5. Identification of an intracoronary thrombus by angiography or autopsy.
6. **Definite myocardial infarction**

Copies of medical records meet with the above requirements and laboratory examination results (ECG, laboratory test report, imaging report, etc.) are provided, or results of the above laboratory tests are clearly shown in medical records including specific description of troponin value, myocardial infarction site shown in ECG or segmental wall movement abnormality by echocardiography, etc.

1. **Probable myocardial infarction**
2. At least one evidence of myocardial ischemia without cTn results;
3. Elevation of cTn shown in laboratory test report or described in the medical records but without typical presentation of myocardial ischemia.
4. **Possible myocardial infarction**
5. Typical symptoms of myocardial ischemia and non-specific laboratory results are described in the medical records (conclusion only but without exact values or specific description);
6. Diagnosis of myocardial infarction is shown on the diagnosis/death certificate but without any other evidence of relevant medical records or laboratory tests;
7. Diagnosis of myocardial infarction that can be clearly described by patients or family members during follow-up visits or over the phone but no data available.

**Ⅲ. Stroke ^1^**

Stroke is defined as presentation of acute vasogenic focal neurological defect with relevant symptoms and signs lasting ≥ 24 hours which could be confirmed by CT or MRI. Cardiovascular death event should also be reported when a patient dies of a stroke. Subarachnoid hemorrhage diagnosed by CT or MRI belongs to stroke after trauma, tumor or [cerebrovascular](javascript:;) malformation causes are excluded.

1. **Definite stroke**

Stroke presented with the above typical symptoms and signs with or without CT/MRI evidence, or a definite ischemic or [hemorrhagic](javascript:;) stroke diagnosed by CT/MRI without typical symptoms and signs is defined as definite stroke.

1. **Possible stroke**

Clinically suspicious stroke without typical symptoms or CT/MRI evidence, or stroke with diagnosis (or the corresponding diagnostic term) shown on the diagnosis/death certificate but without clear medical records, or stroke with typical symptoms and signs described by the patient or family members is defined as possible stroke.

Determination of the type of stroke by CT/MRI within 3 weeks is required.

1. **Hemorrhagic stroke**

Hemorrhagic stroke is defined as when it is clearly diagnosed by CT/MRI. (Attention: major bleeding event should also be reported when hemorrhagic stroke meets the criteria of BARC 3c bleeding).

1. **Ischemic stroke**

Ischemic stroke is defined when it is clearly diagnosed by CT/MRI. (Attention: bleeding transformation of ischemic stroke is still defined as ischemic stroke).

1. **Subarachnoid hemorrhage**

Subarachnoid hemorrhage is defined when it is clearly diagnosed by CT/MRI.

1. **Undetermined stroke**

Stroke without CT/MRI evidence causing difficulty in determination of the clinical type, or stroke diagnosis shown on diagnosis/death certificate only without other relevant medical records or described by the patient or family members is defined as undetermined stroke.

**Ⅳ. Systemic thromboembolism ^3^**

Systemic thromboembolism is defined as acute limb or organ (kidney, mesenteric artery, spleen, retina, or graft) vascular obstruction determined by imaging, surgery, or autopsy, with or without corresponding symptoms or signs.

1. **Definite systemic thromboembolism**

Definite systemic thromboembolism is defined when the above criteria are met.

1. **Probable systemic thromboembolism**

Probable systemic thromboembolism is defined when clinical presentation of corresponding symptoms or signs is present but without relevant imaging, surgical or autopsy records.

1. **Possible systemic thromboembolism**

Possible systemic thromboembolism is defined when the diagnosis is shown on diagnosis/death certificate only but without any relevant medical records.

**Ⅴ. Stent thrombosis ^4^**

1. **Definite stent thrombosis**

Definite stent thrombosis is defined when acute coronary syndrome (ACS) is clinically diagnosed and stent thrombosis is confirmed by angiography or autopsy.

1. **Probable stent thrombosis**

Probable stent thrombosis is defined when unexplained death happened within 30 days after stent implantation, or any MI that is, irrespective of the time after the procedure, related to documented acute ischemia in the corresponding territory of the stented coronary artery without angiographic confirmed stent thrombosis and in the absence of any other obvious cause.

1. **Possible stent thrombosis**

Possible stent thrombosis is defined when any unexplained death occurred from 30 days after stent implantation.

**Ⅵ. Unplanned revascularization ^4^**

1. **Unplanned target vessel revascularization**

Unplanned percutaneous coronary intervention (PCI) or coronary artery bypass grafting (CABG) was performed again in the main coronary artery vessels and the upstream and downstream branches of vessels close to the target lesion (within 5 mm proximal or distal to the stent).

1. **Definite unplanned target vessel revascularization**

Definite unplanned target vessel revascularization is defined as the above criteria is met with evidence of angiographic imaging or intervention reports or detailed description in medical records.

1. **Possible unplanned target vessel revascularization**

Possible unplanned target vessel revascularization is defined as the above criteria is met but without angiographic imaging or intervention reports or detailed description in medical records, or described by the patient or family members.

1. **Unplanned target lesion revascularization**

Unplanned PCI or CABG was performed again in the target lesion (within 5 mm proximal or distal to the stent) due to in-stent restenosis or other complications.

1. **Definite unplanned target lesion revascularization**

Definite unplanned target lesion revascularization is defined as the above criteria is met with evidence of angiographic imaging records or intervention reports or detailed description in medical records.

1. **Possible unplanned target lesion revascularization**

Possible unplanned target vessel revascularization is defined as the above criteria is met but without angiographic imaging or intervention reports or detailed description in the medical records, or described by the patient or family members.

**Safety endpoints**

**Ⅰ. International Society of Thrombosis and Hemostasis (ISTH) bleeding ^5^**

1. **ISTH major bleeding**

As general principles, a definition of major bleeding needs to be based on objective criteria, and major bleeds are those that result in death, are life-threatening, cause chronic sequelae or consume major health-care resources. With this in mind, the Control of Anticoagulation Subcommittee recommends the following criteria for major bleeding in non-surgical patients:

1. Fatal bleeding, and/or
2. Symptomatic bleeding in a critical area or organ, such as intracranial, intraspinal, intraocular, retroperitoneal, intraarticular or pericardial, or intramuscular with compartment syndrome, and/or
3. Bleeding causing a fall in hemoglobin level of 20 g/L (1.24 mmol/L) or more, or leading to transfusion of two or more units of whole blood or red cells.
4. **Definite ISTH major bleeding**

Definite ISTH major bleeding meeting is defined as the above criteria is met, with the copies of medical records including laboratory test reports, examination/treatment records.

1. **Possible ISTH major bleeding**

Possible ISTH major bleeding is defined as the above criteria is met, but without specific medical records or the diagnosis only shown on the diagnosis/death certificate, or described by the patient or family members.

1. **Clinically relevant non-major bleeding (CRNMB)**

A clinically overt bleeding that does not meet the criteria for an ISTH major bleeding but results in ≥ 1 of the following clinical responses:

1. A hospital admission;
2. A physician-guided medical or surgical treatment;
3. A physician-guided change, interruption (more than omitting 1 dose), or discontinuation of the study drug.
4. **Definite CRNMB**

Definite clinically relevant non-major bleeding event is defined as the above criteria is met, with the copies of medical record.

1. **Possible CRNMB**

Possible clinically relevant non-major bleeding event is defined as the above criteria is met, but without specific medical records or the diagnosis only shown on the diagnosis/death certificate, or described by the patient or family members.

**II. BARC bleeding ^6^**

1. **BARC minor bleeding**
2. **Type 0:** No bleeding
3. **Type 1:** Bleeding that is not actionable and does not cause the patient to seek unscheduled performance of studies, hospitalization, or treatment by a healthcare professional; may include episodes leading to self-discontinuation of medical therapy by the patient without consulting a healthcare professional.
4. **Type 2:** Any overt, actionable sign of hemorrhage (e.g., more bleeding than would be expected for a clinical circumstance, including bleeding found by imaging alone) that does not fit the criteria for type 3, 4, or 5 but does meet at least one of the following criteria:
   1. Requiring nonsurgical, medical intervention by a healthcare professional,
   2. Leading to hospitalization or increased level of care, or
   3. Prompting evaluation.
5. **BARC major bleeding**
6. **Type 3**

**Type 3a**

1. Overt bleeding plus hemoglobin drop of 30 to < 50 g/L^*^ (provided hemoglobin drop is related to bleed);
2. Any transfusion with overt bleeding.

**Type 3b**

1. Overt bleeding plus hemoglobin drop ≥ 50 g/L^*^ (provided hemoglobin drop is related to bleed);
2. Cardiac tamponade;
3. Bleeding requiring surgical intervention for control (excluding dental/nasal/skin/hemorrhoid);
4. Bleeding requiring intravenous vasoactive agents.

**Type 3c**

1. Intracranial hemorrhage (does not include microbleeds or hemorrhagic transformation; does include intraspinal);
2. Subcategories confirmed by autopsy, imaging, or lumbar puncture;
3. Intraocular bleed compromising vision.
4. **Type 4: CABG-related bleeding**
5. Perioperative intracranial bleeding within 48 hours;
6. Reoperation after closure of sternotomy for the purpose of controlling bleeding;
7. Transfusion of ≥ 5 U whole blood or packed red blood cells within a 48-hour period^†^;
8. Chest tube output ≥ 2 liters within a 24-hour period.
9. **Type 5: Fatal bleeding**

**Type 5a**

1. Probable fatal bleeding;
2. No autopsy or imaging confirmation but clinically suspicious.

**Type 5b**

1. Definite fatal bleeding;
2. Overt bleeding or autopsy or imaging confirmation

CABG indicates coronary artery bypass graft. Platelet transfusions should be recorded and reported but are not included in these definitions until further information is obtained about the relationship to outcomes. If a CABG-related bleeding is not adjudicated as at least a type 3 severity event, it will be classified as not a bleeding event. If a bleeding event occurs with a clear temporal relationship to CABG (i.e., within a 48-h time frame) but does not meet type 4 severity criteria, it will be classified as not a bleeding event.

^*^Corrected for transfusion (1 U packed red blood cells or 1 U whole blood = 10 g/L hemoglobin).

^†^Cell saver products are not counted.

1. **Definite BARC bleeding**

Definite BARC bleeding is defined as the above criteria is met, with the copies of medical record including laboratory test reports and examination/treatment records.

1. **Possible BARC bleeding**

Possible BARC bleeding is defined as the above criteria is met, but without specific medical record or the diagnosis only shown on the diagnosis/death certificate, or described by the patient or family members.

**Appendix 5. Definition of protocol deviations in OPTIMA-4 trial^7^**

1. Study protocol deviations, including enrollment of participants who do not meet the inclusion criteria or meet the exclusion criteria, without informed consent, use of prohibited drugs, without reports of SAE or study endpoints, and withdrawal from the study;
2. Randomization deviations, including randomization before consent from patients, input of wrong randomization information, and other randomization deviations;
3. Antithrombotic therapy deviations, including use of another antithrombotic therapy regimen in this study or other antithrombotic therapy regimens not used in this study.

Finally, the Events Review Committee will determine major protocol deviations which will influence the scientific integrity of the trial or the safety of the subjects.

**Appendix 6. Prespecified subgroups of interest in OPTIMA-4 trial**

| Items | Subgroups | | |
| --- | --- | --- | --- |
| Age | <75 | ≥75 | - |
| Sex | male | female | - |
| BMI (kg/m^2^) | <28 | ≥28 | - |
| Diabetes | Yes | No | - |
| ACS Diagnosis | UA | NSTEMI | STEMI |
| SYNTAX score | <23 | 23~<32 | ≥32 |
| CHA_2_DS_2_-VASc score | 2-3 | 4-5 | ≥6 |

Abbreviations: BMI, body mass index; ACS, acute coronary syndrome; UA, unstable angina; NSTEMI, non-ST-segment elevation myocardial infarction; STEMI, ST-segment elevation myocardial infarction; SYNTAX, the Synergy between Percutaneous Coronary Intervention with Taxus and Cardiac Surgery; CHA_2_DS_2_-VASc score: Congestive heart failure, Hypertension, Age ≥75 years, Diabetes mellitus, Stroke, Vascular disease, Age 65-74 years, Sex category (female).

**References**

1. Bosch J, Eikelboom JW, Connolly SJ, et al. Rationale, Design and Baseline Characteristics of Participants in the Cardiovascular Outcomes for People Using Anticoagulation Strategies (COMPASS) Trial. *The Canadian journal of cardiology.* 2017;33(8):1027-1035.

2. Thygesen K, Alpert JS, Jaffe AS, et al. Fourth Universal Definition of Myocardial Infarction (2018). *Circulation.* 2018;138(20):e618-e651.

3. Cutlip DE, Windecker S, Mehran R, et al. Clinical end points in coronary stent trials: a case for standardized definitions. *Circulation.* 2007;115(17):2344-2351.

4. Cannon CP, Bhatt DL, Oldgren J, et al. Dual Antithrombotic Therapy with Dabigatran after PCI in Atrial Fibrillation. *The New England journal of medicine.* 2017;377(16):1513-1524.

5. Schulman S, Angerås U, Bergqvist D, Eriksson B, Lassen MR, Fisher W. Definition of major bleeding in clinical investigations of antihemostatic medicinal products in surgical patients. *Journal of thrombosis and haemostasis : JTH.* 2010;8(1):202-204.

6. Mehran R, Rao SV, Bhatt DL, et al. Standardized bleeding definitions for cardiovascular clinical trials: a consensus report from the Bleeding Academic Research Consortium. *Circulation.* 2011;123(23):2736-2747.

7. Branch KRH, Probstfield JL, Bosch J, et al. Total events and net clinical benefit of rivaroxaban and aspirin in patients with chronic coronary or peripheral artery disease: The COMPASS trial. *American heart journal.* 2023;258:60-68.
